# Identification of LSM Family Members as Novel Unfavorable Biomarkers in Hepatocellular Carcinoma

**DOI:** 10.3389/fonc.2022.871771

**Published:** 2022-05-12

**Authors:** Hongkai Zhuang, Bo Chen, Chenwei Tang, Xinming Chen, Wenliang Tan, Lei Yang, Zhiqin Xie, Xiaowu Ma, Qingbin Wang, Chuanzhao Zhang, Changzhen Shang, Yajin Chen

**Affiliations:** ^1^Department of Hepatobiliary Surgery, Sun Yat-sen Memorial Hospital, Sun Yat-sen University, Guangzhou, China; ^2^Guangdong Provincial Key Laboratory of Malignant Tumor Epigenetics and Gene Regulation, Sun Yat-sen Memorial Hospital, Sun Yat-Sen University, Guangzhou, China; ^3^Department of Breast Cancer, Cancer Center, Guangdong Provincial People’s Hospital, Guangdong Academy of Medical Sciences, Guangzhou, China; ^4^Department of Hepatobiliary Surgery, Shenshan Medical Hospital, Sun Yat-Sen Memorial Hospital, Sun Yat-Sen University, Shanwei, China; ^5^Department of General Surgery, Guangdong Provincial People`s Hospital, Guangdong Academy of Medical Sciences, Guangzhou, China

**Keywords:** HCC, LSM, prognosis, ICB, immunosuppression, CD8^+^ T cell

## Abstract

**Background:**

Smith-like (LSM) family members play critical roles in multiple oncologic processes in several types of malignancies. The study on LSM family members of HCC might provide new insights into the tumorigenesis and therapeutic strategies of HCC.

**Methods:**

The clinical significance and oncologic biological functions of LSM family members were assessed through multiple bioinformatics methods and *in vitro* studies. The potential correlation between LSM family members and tumor immunity was also investigated using single sample gene set enrichment analysis (ssGSEA) and the ESTIMATE algorithm.

**Results:**

LSM family member overexpression in HCC was significantly correlated with poor clinical outcomes such as higher TNM stage, advanced histologic grade, and worse prognosis. A risk score system based on LSM5, LSM10, LSM12, and LSM14B showed a reliable predictive ability for OS of HCC patients. Functional enrichment analysis demonstrated that LSM family members overexpressed were all involved in cell cycle related biological processes. Besides, LSM12, LSM14A, and LSM14B were found to be significantly associated with PI3K-Akt-mTOR and T cell receptor signaling pathways. Tumors with LSM12, LSM14A, and LSM14B overexpression exhibited lower infiltration of activated CD8^+^ T cells with declined cytolytic activity and immune score, but increased infiltration of Th2 cells and Th2/Th1. LSM12, LSM14A, and LSM14B overexpression is also associated with higher tumor-related immune checkpoints (e.g., PD-L1, B7-H3, and PVR) expression and increased therapeutic insensitivity to immune checkpoint blockade (ICB). Moreover, the knockdown of LSM12, LSM14A, and LSM14B significantly inhibited the proliferation and invasion of HCC cells.

**Conclusion:**

This study systematically investigated the expression pattern and biological values of LSM family members in HCC and identified LSM family members as novel therapeutic targets in HCC.

## Introduction

Hepatocellular carcinoma (HCC), one of the most prevalent malignancies, ranks as the third leading cause of tumor-related death worldwide, with an overall 5-year survival rate of less than 20% (1, 2). Despite numerous efforts for early diagnosis and novel therapeutic strategies, the survival of HCC patients remains unsatisfactory, due to the specific tumor microenvironment (TME) and tumor heterogeneity in HCC (3–6). Therefore, it is urgent to investigate the molecular mechanisms underlying hepatocarcinogenesis and TME development to design more effective precision treatments for HCC.

The Smith-like (LSM) family consists of 13 members (e.g., LSM1, LSM2, LSM3, LSM4, LSM5, LSM6, LSM7, LSM8, LSM10, LSM11, LSM12, LSM14A, and LSM14B), which are widely known as the RNA-binding protein family, and are generally involved in various cellular biological processes (e.g., RNA-processing tasks and ion mobilizations) (7, 8). For example, the LSM1-7 complex functions in cytoplasmic mRNA decay through interaction with mRNA degradative factors (9). Furthermore, the LSM2-8 complex in the nucleus functions in pre-mRNA splicing by interacting with the U6 snRNA (10). Additionally, a previous study by Zhang et al. also identified LSM12 as an NAADP receptor essential for NAADP-evoked TPC activation and Ca2^+^ transportation from intracellular acidic stores (11). The oncologic roles of LSM family members have also been identified in several tumor types (12–14). LSM1 overexpression contributes to a more advanced malignant phenotype in pancreatic cancer (12). Moreover, Watson et al. also identified the essential role of LSM1 overexpression in tumor progression in lung cancer and mesothelioma (13). Besides, it is also reported that LSM4 knockdown inhibits tumor proliferation, invasion, and glycolysis metabolism in ovarian cancer (14). However, the underlying mechanism of LSM family members in carcinogenesis still remains poorly understood, especially for HCC. Therefore, it is of great significance to explore the potential biological roles of LSM family members in tumorigenesis and TME development in HCC.

Through public databases and multiple bioinformatics analyses, we comprehensively investigated the expression patterns and clinical significance of LSM family members in HCC. Additionally, with the help of gene set variation analysis, we are also the first to investigate the underlying biological functions of LSM family members in hepatocarcinogenesis and tumor immunity.

## Materials and Methods

### RNA Information Acquisition

Publicly available mRNA data and corresponding clinical information of HCC patients were downloaded from the Cancer Genome Atlas (TCGA, https://cancergenome.nih.gov/). Of the 371 HCC cases in the TCGA HCC cohort, 343 had an OS of >1 month. Besides, the HCC cohort from the International Cancer Genome Consortium (ICGC, https://icgc.org/) database was also used for expression pattern and prognostic value validation for LSM family members. The TCGA and ICGC HCC cohorts are both available freely as public databases, for which local ethics approval was not needed.

### LSM Family Member Expression Analysis

First, the expression differences of LSM family members between HCC tissues (n = 50) and the corresponding adjacent normal tissues (n = 50) were evaluated using the TCGA HCC cohort. Then, the ICGC HCC cohort was also used to evaluate the expression differences of LSM family members between HCC tissues (n = 231) and adjacent normal tissues (n = 201). Furthermore, LSM family member expression in various TNM stages and histologic grades was evaluated using the Kruskal–Wallis test or Wilcoxon test.

### Prognostic Values of LSM Family Members

A Kaplan–Meier survival analysis was conducted to investigate the prognostic value [e.g., overall survival (OS) and disease-free survival (DFS)] of LSM family members in HCC. Additionally, using the R package ‘glmnet’, the critical prognosis-associated LSM family members were further determined through the least absolute shrinkage and selection operator (Lasso) regression analysis. Then, a risk score system for OS was established as the following formula: risk score = ∑coef (LSM_i_) × expr (LSM_i_). For external validation, using the same multivariable Cox regression coefficients obtained in the TCGA HCC cohort, the risk score was further calculated for HCC cases in the ICGC HCC cohort. Using the R package ‘survminer’, KM survival analysis was also performed according to the optimal cutoff point from X-tile (version 3.6.1) (15). The predictive performance of the risk score system was evaluated using the AUC values of the ROC curves and C-index. To verify whether the risk score system could be used as an independent prognostic factor, we conducted univariate and multivariate Cox regression analyses for clinicopathological characteristics (e.g., TNM stage and histologic grade) and the risk score system.

### Functional Enrichment Analysis

A gene set variation analysis (GSVA) was used to calculate the enrichment scores of specific gene sets for each sample based on RNA-seq (16). Using the R package ‘gsva’, we performed GSVA to estimate the enrichment level of 13 critical ontology gene sets obtained from the Molecular Signatures Database (MSigDB, http://software.broadinstitute.org/gsea/msigdb) in the TCGA HCC cohort, namely, DNA repair, G2M checkpoint, E2F targets, mitotic spindle, glycolysis, hedgehog signaling, mTORC1 signaling, MYC targets V1, MYC targets V2, Notch signaling, PI3K-AKT-mTOR signaling, TGF-β signaling, and WNT β-catenin signaling. Then, the correlation between the expression of LSM family members and these critical oncogenic biological processes was assessed by the Pearson correlation analysis (|Cor| >0.3, P-value <0.05). Additionally, significant co-expressed genes of LSM family members were figured out by the Pearson correlation analysis (|Cor| ≥0.55 and P-value <0.05). Then, these co-expressed genes of LSM family members were imported into ConsensusPathDB (http://cpdb.molgen.mpg.de/) for Kyoto Encyclopedia of Genes and Genomes (KEGG) analysis; P <0.05 was considered statistically significant.

### T-Cell Infiltration in HCC

GSVA was also performed to quantify the enrichment levels of T cell-related terms extracted from previous studies, namely, activated CD8+ T cell, regulatory T cell (Treg cell), type 1 T helper cell (Th1 cell), type 2 T helper cell (Th2), Th2/Th1, T follicular helper cell, and cytolytic activity (17, 18). Then, correlation analysis between LSM family members and these T cell-related terms in HCC was assessed with |Pearson correlated coefficient| >0.2 and a P-value of <0.05. We also evaluated the differences in these T cell-related terms between high- and low-LSM family member expression groups. Additionally, using the R package ‘ESTIMATE’, we performed the ESTIMATE algorithm to generate an immune score, the higher of which suggested a higher level of anti-tumor immunity in tumor tissues (19). Then, we investigated the difference in immune scores between low- and high-LSM family member expression groups.

### Cell Cultural and Transfection

Human SNU-387 cells, purchased from the Cell Bank of the Chinese Academy of Sciences (Shanghai, China), were cultured with Dulbecco’s Modified Eagle Medium (DMEM; Gibco BRL, USA), and supplied with 10% fetal bovine serum (FBS; Procell Life Science & Technology Co., Ltd., Wuhan, China) in an incubator with 5% CO2 at 37 °C. The small interfering RNAs (si-RNA) of LSM12, LSM14A, and LSM14B (si-LSM12#1/2; si-LSM14A#1/2; si-LSM14B#1/2) were obtained from RiboBio (Guangzhou, China). In short, cells were seeded in 6-well plates and cultured to an appropriate confluence, then transfected with different si-RNAs by Lipofectamine 3000 (Thermo Fisher Scientific, USA) according to its protocol. After 48 h incubation, the transfected cells were collected for further analysis. The efficacy of si-LSM12/14A/14B RNAs in SNU-387 cells was assessed using qRT-PCR. The target sequences of LSM12, LSM14A, and LSM14B siRNAs were as follows: si-LSM12#1: 5`-GCAGACATCTTGCTCATAA-3`; si-LSM12#2: 5`-GAGCCATGTACGCAAAATA-3`; si-LSM14A#1: 5`-GAGCAACTCAGAAATGATA-3`; si-LSM14A#2: 5`-GGAGAAGCCTGTAAATGGT-3`; si-LSM14B#1: 5`-GAAGACCGTCCCACAGATA-3`; si-LSM14B#2: 5`-CGACAACATCTCTTCTGAA-3`. Furthermore, the PCR primers were as follows: LSM12, 5`-TCTTCCAGTGGAAAGCCCAAC-3` (forward), 5`-GCTTTGCTGGCAAGCTTACT-3` (reverse); LSM14A, 5`-TGTGATGACAATAGAGAACGGAGA-3` (forward), 5`-AAGGTACCACCTCTGCCAC-3` (reverse); LSM14B, 5`-CTGAACTCAAGACCAGCTCCAG-3` (forward), 5`-GAACTGCGGCCACGAAGAA-3` (reverse); GAPDH forward, 5`-TGACTTCAACAGCGACACCCA-3`; GAPDH reverse, and 5`-CACCCTGTTGCTGTAGCCAAA-3`.

### Cell Counting Kit-8 (CCK-8) Assay

Cell viability was examined using the Cell Counting Kit-8 (APExBIO, USA). In short, SNU-387 cells transfected with different small interfering RNAs for LSM12, LSM14A, and LSM14B (LSM12: si-NC, si-LSM12#1, si-LSM12#2; LSM14A: si-NC, si-LSM14A#1, LSM14A#2; LSM14B: si-NC, si-LSM14B#1, si-LSM14B#2) were seeded into 96-well plates at a density of 2 × 10^3^. At 1, 2, 3, 4, 5, and 6 days, the mix solution with 10 µl CCK-8 reagent and 100 µl DMEM was added to each well and re-incubated for 3 h, followed by the absorbance at 450 nm measured by a microplate reader (Thermo MK3, Thermo Fisher Scientific, USA).

### Transwell Invasion Assays

Transfected cells suspended in serum-free medium were seeded in triplicate into each of the upper transwell chambers (Corning, USA) with Matrigel (Corning, USA) at a density of 1 × 10^4^ and incubated in 200 µl DMEM with free serum. Furthermore, the lower chambers contained 500 µl DMEM supplemented with 100 µl FBS. Then cells from the upper compartments were wiped out after 24 h, and the invaded cells were fixed with paraformaldehyde for 15 min, followed by staining with 0.3% crystal violet for 15 min at room temperature. Cell counting was conducted using microscopy.

### Statistical Analysis

All statistical analyses were conducted using the R software 4.0.5 (http://r-project.org/) and GraphPad Prism 8.0 software (GraphPad Software, Inc.). Group difference analyses were conducted using the Kruskal–Wallis test or Wilcoxon test and expressed as mean ± standard deviation (SD). Correlation analyses were performed using the Pearson correlation coefficient. Moreover, a P-value of < 0.05 was considered statistically significant.

## Results

### Overexpression of LSM Family Members in HCC

**Figure 1:**
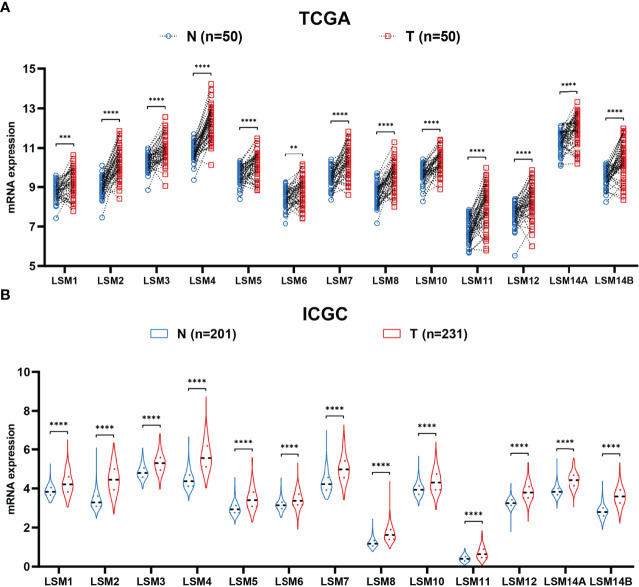
Differential expression analysis of LSM family members. **(A)** LSM family members were significantly upregulated in HCC tissues compared with that in the adjacent non-tumor tissues in the TCGA HCC cohort. **(B)** LSM family members were significantly up-regulated in HCC tissues compared with that in the adjacent non-tumor tissues in the ICGC HCC cohort. LSM, Smith-like (LSM) family members; HCC, hepatocellular carcinoma; TCGA, the Cancer Genome Atlas; ICGC, the International Cancer Genome Consortium. **P-value <0.01; ***P-value <0.001; ****P-value <0.0001.

Firstly, the paired differential expression analysis based on the TCGA HCC cohort demonstrated that LSM family members were all notably overexpressed in HCC tissues compared to adjacent normal tissues (**Figure 1A**). Additionally, the ICGC HCC cohort also revealed that all LSM family members were significantly upregulated in HCC tissues compared with adjacent normal tissues (**Figure 1B**). Taken together, these results suggest the overexpression of LSM family members in HCC.

### LSM Family Members in Various TNM Stage and Histologic Grade

**Figure 2:**
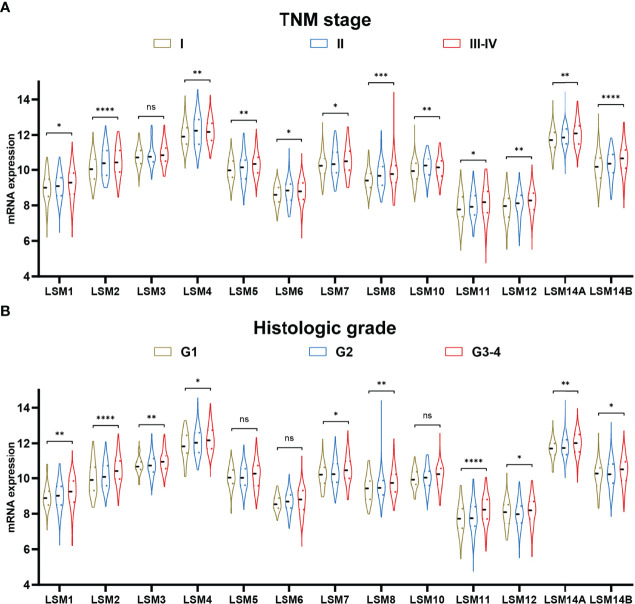
LSM family member expression in TNM stage and histologic grade. **(A)** Higher expression of LSM1, LSM2, LSM4, LSM5, LSM6, LSM7, LSM8, LSM10, LSM11, LSM12, LSM14A, and LSM14B were observed in tumors with advanced TNM stage. **(B)** Higher expression of LSM1, LSM2, LSM3, LSM4, LSM7, LSM8, LSM11, LSM12, LSM14A, and LSM14B were also observed in tumors with higher histologic grade. LSM, Smith-like (LSM) family members. ns >0.05; *P-value <0.05; **P-value <0.01; ***P-value <0.001; ****P-value <0.0001.

Twelve LSM family members (e.g., LSM1, LSM2, LSM4, LSM5, LSM6, LSM7, LSM8, LSM10, LSM11, LSM12, LSM14A, and LSM14B) were significantly associated with advanced TNM stage (**Figure 2A**). Besides, higher expressions of ten LSM family members (e.g., LSM1, LSM2, LSM3, LSM4, LSM7, LSM8, LSM11, LSM12, LSM14A, and LSM14B) were also observed in tumors with higher histological grade, though not statistically significant for LSM5, LSM6, and LSM10 (**Figure 2B**). Taken together, these findings suggest that LSM family member overexpression may be associated with tumor progression in HCC.

### The Prognostic Value of LSM Family Members in HCC

**Figure 3:**
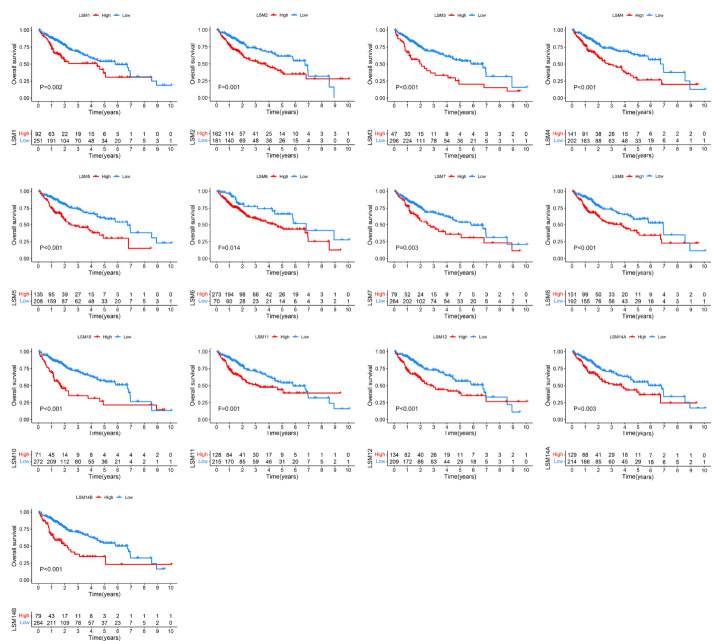
KM survival curves for OS of LSM family members in HCC. KM, Kaplan–Meier; OS, overall survival; LSM, Smith-like (LSM) family members.

**Figure 4:**
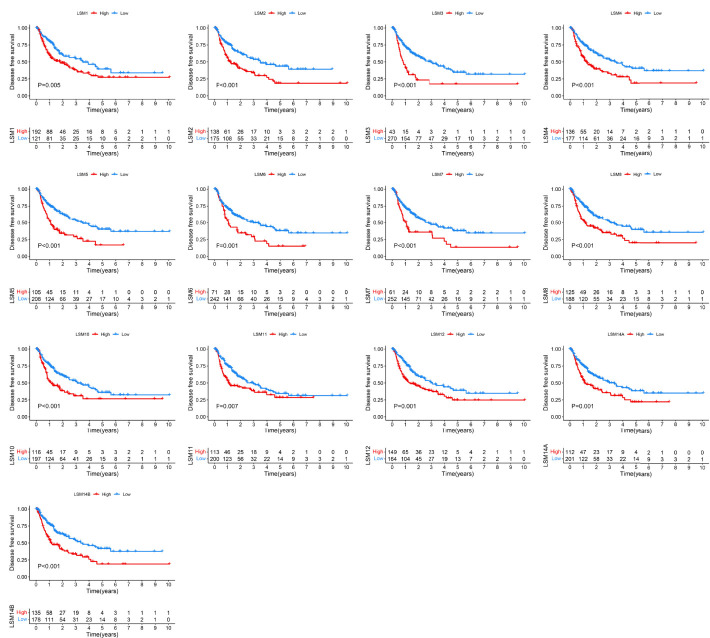
KM survival curves for DFS of LSM family members in HCC. KM, Kaplan–Meier; DFS, disease-free survival; LSM, Smith-like (LSM) family members; HCC, hepatocellular carcinoma.

**Figure 5:**
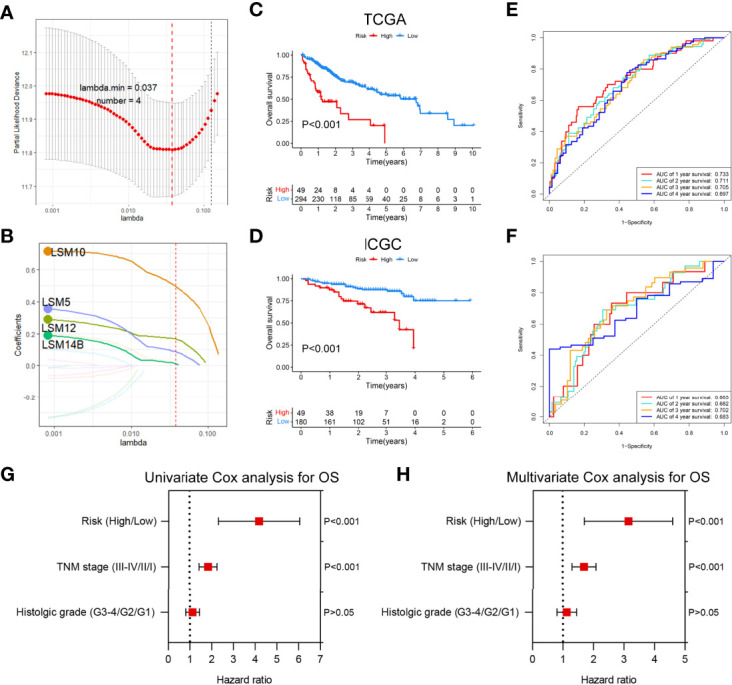
Development and validation of a risk score system for OS based on LSM family members. **(A, B)** Lasso regression analysis identified five critical prognosis-associated LSM family members (e.g., LSM5, LSM10, LSM12, and LSM14B) for HCC. **(C, D)** KM survival curves for OS of patients with HCC according to the risk scores in the TCGA HCC and ICGC HCC cohorts. **(E, F)** ROC curve analysis of the risk score system for 1-, 2-, 3-, and 4-year OS prediction in the TCGA HCC and ICGC HCC cohorts. **(G)** Univariate Cox regression analysis demonstrated that the risk score system and TNM stage were prognostic factor for OS of patients with HCC. **(H)** Multivariate Cox regression analysis demonstrated that the risk score system was an independent unfavorable prognostic factor for OS of patients with HCC. OS, overall survival; LSM, Smith-like (LSM) family members; Lasso, least absolute shrinkage and selection operator regression analysis; HCC, hepatocellular carcinoma; KM, Kaplan–Meier; TCGA, the Cancer Genome Atlas; ICGC, the International Cancer Genome Consortium; ROC, the receiver operating characteristic curve.

To determine the prognostic values of LSM family members in patients with HCC, we assessed the associations between the mRNA expression of LSM family members and OS or DFS using KM survival analysis. KM survival analysis demonstrated that LSM family members were all notably associated with shorter OS or DFS of HCC patients (**Figures 3**, **4**). Additionally, four critical prognosis-associated LSM family members, namely, LSM5, LSM10, LSM12, and LSM14B, were identified using Lasso regression analysis (**Figures 5A, B**). Then, a risk score system based on these four critical prognosis-associated LSM family members was established using multivariate correlation analysis. Risk score = 0.1435 × LSM5 expression + 06358 × LSM10 expression + 0.2092 × LSM12 expression + 0.1019 × LSM14B expression. The KM survival curves for OS demonstrated that patients with low-risk scores had superior OS compared with those with high-risk scores (**Figure 5C**). For validation, risk scores were also calculated for 229 patients with HCC in the ICGC HCC cohort. Furthermore, similar results of KM survival analysis were also observed in the ICGC HCC cohort (**Figure 5D**). The C-indexes of the risk score system for OS prediction in the TCGA and ICGC cohorts were 0.676 (95% CI, 0.621–0.731) and 0.658 (95% CI, 0.571–0.744), respectively, suggesting a reliable predictive capability of the risk score system. Additionally, in the TCGA HCC cohort, the AUC values of the risk score system for 1-, 2-, 3-, and 4-year OS prediction were 0.733, 0.711, 0.705, and 0.697, respectively (**Figure 5E**). Consistently, the AUC values of the risk score system for 1-, 2-, 3-, and 4-year OS predictions were 0.683, 0.682, 0.702, and 0.683 in the ICGC HCC cohort (**Figure 5F**). Moreover, univariate and multivariate Cox analyses identified the risk score system as an independent unfavorable prognostic parameter in HCC (**Figures 5G, H**). These results proved the significant prognostic value of LSM family members in HCC.

### Analysis of Biological Pathways in LSM Family Members

**Figure 6:**
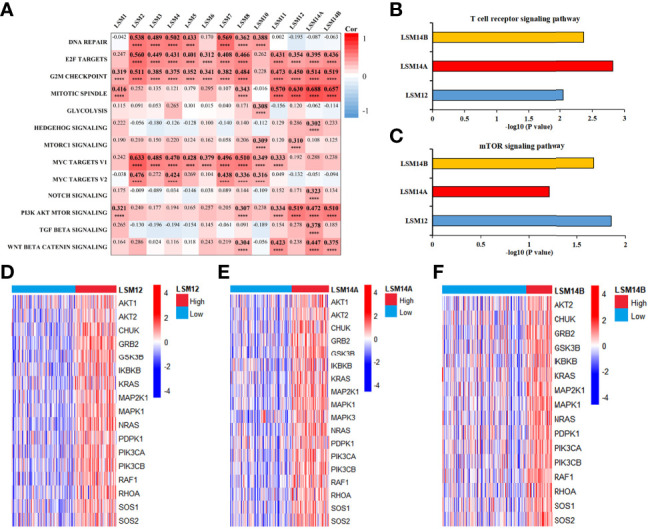
Functional enrichment analysis for LSM family members in HCC. **(A)** Heat map to show the correlation between LSM family and critical biological pathway. **(B)** KEGG analysis demonstrated that LSM12, LSM14A, and LSM14B may play important role in T cell receptor signaling pathway in HCC. **(C)** KEGG analysis also identified the underlying interactions between the mTOR signaling pathway and LSM12, LSM14A, and LSM14B. **(D–F)** Heat maps to show the overlapped co-expressed genes involved in T-cell receptor signaling pathway and mTOR signaling pathway for LSM12, LSM14A, and LSM14B, respectively. LSM, Smith-like (LSM) family members; KEGG, Kyoto Encyclopedia of Genes and Genomes analysis; HCC, hepatocellular carcinoma. ****P-value <0.0001.

Correlation analyses between LSM family members and thirteen critical biological pathways are shown in **Figure 6A**. Interestingly, all LSM family members but LSM10 were significantly associated with cell cycle related biological processes, such as the G2M checkpoint, E2F targets, and mitotic spindle. Nine LSM family members, including LSM2-8, LSM10, and LSM11, were notably associated with MYC signaling pathways. Then, six LSM family members may play crucial roles in PI3K-Akt-mTRO signaling in HCC, namely, LSM1, LSM8, LSM11, LSM12, LSM14A, and LSM14B. Besides, four LSM family members (e.g., LSM8, LSM11, LSM14A, and LSM14B) were significantly associated with WNT β-catenin signaling in HCC. Additionally, co-expression genes of LSM family members (|Pearson correlated coefficient| ≥0.55 and P-value <0.05) were imported into ConsensusPathDB for KEGG analysis, which is shown in **Table S1**. Interestingly, LSM12, LSM14A, and LSM14B were observed to play critical roles in the T-cell receptor signaling pathway, suggesting their potential effects on tumor immunity in HCC (**Figure 6B**). Of note, KEGG analysis also consistently identified the underlying interactions between the mTOR signaling pathway and LSM12, LSM14A, and LSM14B (**Figure 6C**). Then, the overlapped co-expressed genes involved in the mTOR signaling pathway and the T-cell receptor signaling pathway were also presented in heat maps for LSM12, LSM14A, and LSM14B, respectively (**Figures 6D–F**).

### LSM12, LSM14A, and LSM14B Overexpression Correlated With Immune Suppression in HCC

**Figure 7:**
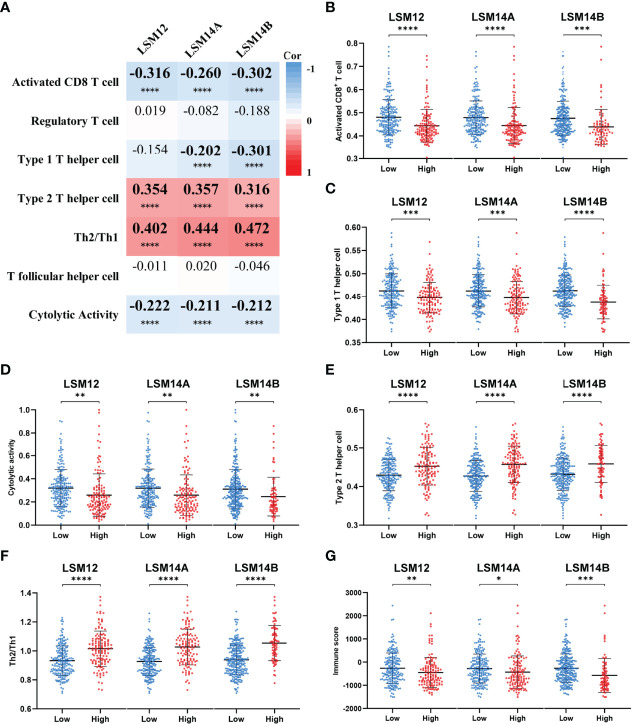
Tumors with LSM12, LSM14A, and LSM14B overexpression were associated with an immunosuppressive phenotype. **(A)** Heat map to show the association between LSM12, LSM14A, and LSM14B expression and T-cell-related terms. **(B)** Tumors with higher LSM12, LSM14A, and LSM14B expression exhibited decreased activated CD8^+^ T-cell infiltration compared with those with lower LSM12, LSM14A, and LSM14B expression. **(C)** Tumors with higher LSM12, LSM14A, and LSM14B expression exhibited decreased Th1 cell infiltration compared with those with lower LSM12, LSM14A, and LSM14B expression. **(D)** Tumors with higher LSM12, LSM14A, and LSM14B expression exhibited decreased cytolytic activity compared with those with lower LSM12, LSM14A, and LSM14B expression. **(E)** Tumors with higher LSM12, LSM14A, and LSM14B expression exhibited increased Th2 cell infiltration compared with those with lower LSM12, LSM14A, and LSM14B expression. **(F)** Tumors with higher LSM12, LSM14A, and LSM14B expression exhibited increased Th2/Th1 compared with those with lower LSM12, LSM14A, and LSM14B expression. **(G)** Tumors with higher LSM12, LSM14A, and LSM14B expression exhibited decreased immune score compared with those with lower LSM12, LSM14A, and LSM14B expression. Th1, type 1 T helper cell; Th2 cell, type 2 T helper cell. *P-value <0.05; **P-value <0.01; ***P-value <0.001; ****P-value <0.0001.

Considering that the T-cell receptor signaling pathway was significantly enriched for LSM12, LSM14A, and LSM14B, we further investigated the association between T-cell-related terms and LSM12, LSM14A, and LSM14B expressions. The LSM12, LSM14A, and LSM14B expressions were negatively correlated with the enrichment levels of activated CD8^+^ T cell and cytolytic activity, but positively correlated with the enrichment levels of Th2 cell and Th2/Th1 (**Figure 7A**). Furthermore, tumors with higher expression of LSM12, LSM14A, or LSM14B had lower infiltration of activated CD8^+^ T cell and impaired cytolytic activity, but increased infiltration of Th2 cell and higher Th2/Th1 (**Figures 7B–F**). Of note, through the ESTIMATE algorithm, a lower immune score was observed in tumors with higher expression of LSM12, LSM14A, and LSM14B (**Figure 7G**).

**Figure 8:**
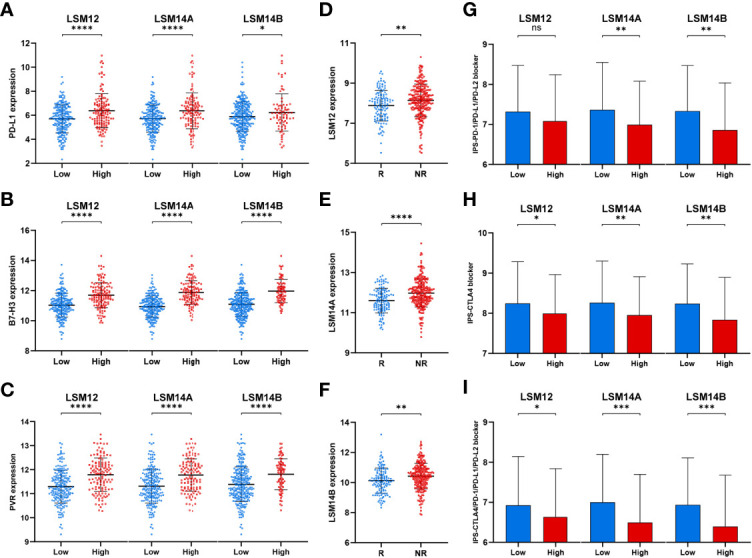
Tumors with higher LSM12, LSM14A, and LSM14B expression exhibited higher expression of tumor-related immune checkpoints and increased therapeutic insensitivity to immune checkpoint blockade. **(A–C)** Differential expression analysis of tumor-related immune checkpoints (e.g., PD-L1, B7-H3, and PVR) between high- and low-expression groups of LSM12, LSM14A, and LSM14B, respectively. **(D–F)** Higher expression of LSM12, LSM14A, and LSM14B were observed in ICB-non-response tumors. **(G)** Tumors with higher LSM14A and LSM14B expression exhibited lower IPS-PD-1/PD-L1/PD-L2. **(H)** Tumors with higher LSM12, LSM14A, and LSM14B expression exhibited lower IPS-CTLA. (**I**) Tumors with higher LSM12, LSM14A, and LSM14B expression exhibited lower IPS-CTLA4/PD-1/PD-L1/PD-L2. ICB, immune checkpoint blockade; NR, non-response; R, response. *P-value <0.05; **P-value <0.01; ***P-value <0.001; ****P-value <0.0001.

We also found that tumors with higher expression of LSM12, LSM14A, and LSM14B had higher expression of tumor-related immune checkpoints (e.g., PD-L1, B7-H3, and PVR), which are important for the immunosuppressive phenotype in malignancies (**Figures 8A–C**) (20–24). Furthermore, ImmuneCellAI (http://bioinfo.life.hust.edu.cn/ImmuCellAI/#!/) was used to predict the therapeutic response to immune checkpoint blockade (ICB). Then, higher expression of LSM12, LSM14A, and LSM14B were observed in ICB-non-response tumors (**Figures 8D–F**). To further verify the association between the response of patients to ICB and the expressions of LSM12, LSM14A, and LSM14B, we evaluated the differences in immunophenoscore (IPS) between different LSM12, LSM14A, and LSM14B expression levels, which were obtained from the Cancer Immunome Atlas (https://tcia.at/) (25). Three subtypes of IPS values (IPS-CTLA4, IPS-PD-1/PD-L1/PD-L2, and IPS-CTLA4/PD-1/PD-L1/PD-L2) were selected to assess the response of HCC patients to anti-PD-1/PD-L1/PD-L2 therapy, anti-CTLA4 therapy, or anti- PD-1/PD-L1/PD-L2 and anti-CTLA4 combination therapy. The IPS-PD-1/PD-L1/PD-L2 was notably higher in tumors with lower expression of LSM14A and LSM14B, but not significantly for LSM12 (**Figure 8G**). Moreover, tumors with lower expression of LSM12, LSM14A, and LSM14B all exhibited increased IPS-CTLA and IPS-CTLA4/PD-1/PD-L1/PD-L2 (**Figures 8H, I**). These results indicate that overexpression of LSM12, LSM14A, and LSM14B might be correlated with immunotolerance and evasion and therapeutic resistance to ICB in HCC.

### LSM12, LSM14A, and LSM14B Knockdown Inhibits the Proliferation and Invasion of HCC Cells

**Figure 9:**
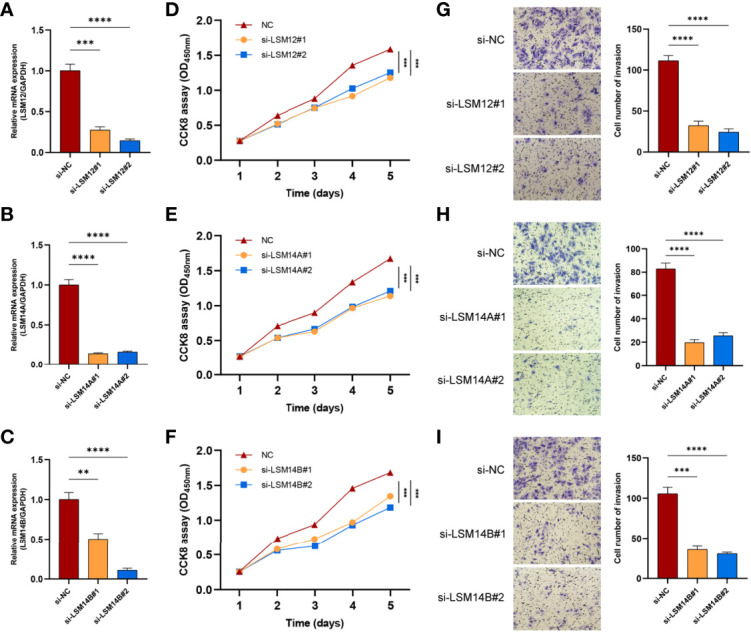
The knockdowns of LSM12, LSM14A, and LSM14B suppress the proliferation and invasion of HCC cells. **(A–C)** qRT-PCR analysis evaluated the efficacies of si-LSM12/14A/14B RNAs in SNU-387 cell. **(D–F)** Assessment of tumor cell proliferation using the CCK8 assay. **(G–I)** Assessment of the invasive capacity of SNU-387 cells using transwell invasion assay. **P-value <0.01; ***P-value <0.001; ****P-value <0.0001.

To further investigate the underlying role of LSM12, LSM14A, and LSM14B in tumor cell proliferation and invasion, we transfected SNU-387 cells with si-LSM12, si-LSM14A, and si-LSM14B RNAs. Moreover, LSM12, LSM14A, and LSM14B expressions were significantly downregulated in the si-RNA groups (P <0.0001) (**Figures 9A–C**). The CCK-8 assay demonstrated that tumor cell proliferation was notably inhibited in LSM12, LSM14A, and LSM14B depleted SNU-387 cells (P <0.0001) (**Figures 9D–F**). Furthermore, the transwell invasion assay demonstrated that the number of invading cells was significantly decreased in the si-RNAs groups compared with those in the NC group (P <0.0001) (**Figures 9G–I**).

## Discussion

These studies showed that overexpression of LSM family members was significantly correlated with advanced TNM stage, histological grade, and worse survival. Additionally, we also constructed a risk score system based on LSM5, LSM10, LSM12, and lsm14B, which was also identified as an independent prognostic factor with optimal predictive ability with reliable C-indexes [0.676 (95% CI, 0.621–0.731) in the TCGA HCC cohort and 0.658 (95% CI, 0.571–0.744) in the ICGC HCC cohort]. These results suggest that LSM family members could serve as novel unfavorable prognostic biomarkers for patients with HCC.

Functional enrichment analysis demonstrated that all LSM family members might take part in the regulation of the cell cycle in HCC. Consistently, suppression of LSM1 expression results in tumor cell proliferation block with decreased G1-phase (26). Fraser et al. also reported that LSM1 knockdown leads to a cytostatic block of the cell cycle (27). Besides, Mili et al. reported that LSM14A interacts directly with tubulin, which is implicated in the stabilization of the mitotic spindle (28). Therefore, it is quite likely that targeting LSM family members may block tumor proliferation through cell cycle arrest in HCC, which deserves further experimental studies.

KEGG analysis revealed that LSM12, LSM14A, and LSM14B were associated with the T-cell receptor signaling pathway, indicating their possible biological roles in T-cell infiltration into the TME of HCC. Additionally, ssGSEA demonstrated that LSM12, LSM14A, and LSM14B overexpression significantly correlated with declined activated CD8^+^ T-cell infiltration and impaired cytolytic activity, but increased infiltration of Th2 cells and higher Th2/Th1. Previous studies have demonstrated that Th2 cells have been widely considered tumor-promoting immune cells, leading to unfavorable clinical outcomes for HCC (29, 30). Besides, a higher level of Th2 cytokines (e.g., IL4 and IL10) are observed in metastatic HCC, whereas Th1 cytokines (e.g., IFN-γ and IL1) are notably decreased (31). Furthermore, IL-4 and IL-10 restrain IFN-γ production and impair cytotoxic cell-mediated anti-tumor immunity, suggesting that an increased shift from Th1 cells to Th2 cells promotes tumor progression and immunosuppression (31–34). Of note, higher expression of tumor-related immune checkpoints (e.g., PD-L1, B7-H3, and PVR) was also observed in tumors with LSM12, LSM14A, and LSM14B overexpression. Of note, LSM12, LSM14A, and LSM14B overexpression was observed in patients non-responsive to ICB. Moreover, tumors with LSM12, LSM14A, and LSM14B overexpression exhibited decreased IPS to ICB. These results further suggested the potential functions of LSM12, LSM14A, and LSM14B in promoting immunotolerance and evasion in HCC.

This study also found that LSM12, LSM14A, and LSM14B overexpression significantly correlated with higher enriched level of the PI3K-Akt-mTOR signaling pathway. Tumors with LSM12, LSM14A, and LSM14B overexpression also exhibited notably higher expression of critical molecules overlapped between the mTOR signaling pathway and T-cell receptor signaling pathway, namely, AKT2, CHUK, GRB2, GSK3B, IKBKB, KRAS, MAP2K1, MAPK1, NRAS, PDPK1, PIK3CA, PIK3CB, RAF1, RHOA, SOS1, and SOS2, which may be the potential targets of LSM12, LSM14A, and LSM14B for tumor immunity in HCC. It has been shown that the activation of the mTOR signaling pathway is associated with PD-L1 overexpression at both levels of mRNA expression and protein translation in multiple malignancies (35). Suppression of the PI3K-Akt-mTOR signaling pathway cannot only restrain tumor growth itself, but also modulates anti-tumor cytokine production and increase-activated CD8^+^ T-cell infiltration (35, 36). Besides, Liu et al. also reported that the inhibition of mTOR signaling by tubermoside-1 decreases PD-L1 expression and enhances anti-tumor immunity (37). Additionally, we preliminarily demonstrated knockdown of LSM12, LSM14A, and LSM14B significantly inhibited tumor cell proliferation and invasion. Taken together, these findings suggest the critical pro-tumor effects of LSM12, LSM14A, and LSM14B overexpression on HCC tumor cell proliferation and metastasis. But in depth experimental works are supposed to be done to demonstrate the underlying mechanisms of LSM12, LSM14A, and LSM14B in hepatocellular carcinogenesis and immunosuppression.

As far as we are aware, this study is the first to systematically describe the expression patterns, clinical significance, and underlying biological functions of LSM family members in HCC. Additionally, for the first time, we proposed that LSM12, LSM14A, and LSM14B overexpression may promote immunotolerance and evasion in HCC. However, several limitations should be acknowledged in this study. First, the prognostic prediction capability of the risk score system should be evaluated in a multi-centered and large cohort. Second, further *in vivo* studies should be performed to investigate the underlying mechanisms by which LSM family members promote tumor progression and immune suppression in HCC.

In summary, our study demonstrated the overexpressed patterns and unfavorable prognostic values of LSM family members in HCC. Of note, we identified the potential inhibition effects of LSM12, LSM14A, and LSM14B on tumor immunity, which laid a novel foundation to further explore the role of LSM family members as novel potential therapeutic targets in HCC.

## Data Availability Statement

The raw data supporting the conclusions of this article will be made available by the authors, without undue reservation.

## Author Contributions

Conceptualization: HZ, YC, CS, and CZ. Methodology: HZ, BC, CT, and XC. Investigation: HZ, BC, CT, and XC. Writing—Original Draft: HZ. Writing—Review and Editing: HZ, BC, CT, XC, WT, LY, ZX, XM, and QW. Visualization: HZ and BC. Supervision: HZ, YC, CS, and CZ. Funding Acquisition: YC, CS, and LW. All authors listed have made a substantial, direct, and intellectual contribution to the work and approved it for publication.

## Funding

This work was supported by grants from the National Natural Science Foundation of China (Nos. 81972263, 82103221, 82072714), the program of Guangdong Provincial Clinical Research Center for Digestive Diseases (2020B1111170004), and the China Postdoctoral Science Foundation (2020M683094).

## Conflict of Interest

The authors declare that the research was conducted in the absence of any commercial or financial relationships that could be construed as a potential conflict of interest.

## Publisher’s Note

All claims expressed in this article are solely those of the authors and do not necessarily represent those of their affiliated organizations, or those of the publisher, the editors and the reviewers. Any product that may be evaluated in this article, or claim that may be made by its manufacturer, is not guaranteed or endorsed by the publisher.

## References

[1] Villanueva A. Hepatocellular Carcinoma. N Engl J Med (2019) 380:1450–62. doi: 10.1056/NEJMra1713263 30970190

[2] Nault JC Villanueva A. Biomarkers for Hepatobiliary Cancers. Hepatology (2021) 73 Suppl 1:115–27. doi: 10.1002/hep.31175 32045030

[3] Bruix J da Fonseca LG Reig M. Insights Into the Success and Failure of Systemic Therapy for Hepatocellular Carcinoma. Nat Rev Gastroenterol Hepatol (2019) 16:617–30. doi: 10.1038/s41575-019-0179-x 31371809

[4] Farzaneh Z Vosough M Agarwal T Farzaneh M. Critical Signaling Pathways Governing Hepatocellular Carcinoma Behavior; Small Molecule-Based Approaches. Cancer Cell Int (2021) 21:208. doi: 10.1186/s12935-021-01924-w 33849569 PMC8045321

[5] Gao Q Wang XY Zhou J Fan J. Heterogeneity of Intermediate-Stage HCC Necessitates Personalized Management Including Surgery. Nat Rev Clin Oncol (2015) 12:10. doi: 10.1038/nrclinonc.2014.122-c1 25421283

[6] Gao Q Wang XY Zhou J Fan J. Multiple Carcinogenesis Contributes to the Heterogeneity of HCC. Nat Rev Gastroenterol Hepatol (2015) 12:13. doi: 10.1038/nrgastro.2014.6-c1 25421581

[7] Mura C Phillips M Kozhukhovsky A Eisenberg D. Structure and Assembly of an Augmented Sm-Like Archaeal Protein 14-Mer. Proc Natl Acad Sci U S A (2003) 100:4539–44. doi: 10.1073/pnas.0538042100 PMC404694 12668760

[8] Fischer S Benz J Spath B Maier LK Straub J Granzow M. The Archaeal Lsm Protein Binds to Small RNAs. J Biol Chem (2010) 285:34429–38. doi: 10.1074/jbc.M110.118950 PMC2966057 20826804

[9] Tharun S He W Mayes AE Lennertz P Beggs JD Parker R. Yeast Sm-Like Proteins Function in mRNA Decapping and Decay. Nature (2000) 404:515–8. doi: 10.1038/35006676 10761922

[10] Achsel T Brahms H Kastner B Bachi A Wilm M Luhrmann R. A Doughnut-Shaped Heteromer of Human Sm-Like Proteins Binds to the 3'-End of U6 snRNA, Thereby Facilitating U4/U6 Duplex Formation *In Vitro* . EMBO J (1999) 18:5789–802. doi: 10.1093/emboj/18.20.5789 PMC1171645 10523320

[11] Zhang J Guan X Shah K Yan J. Lsm12 is an NAADP Receptor and a Two-Pore Channel Regulatory Protein Required for Calcium Mobilization From Acidic Organelles. Nat Commun (2021) 12:4739. doi: 10.1038/s41467-021-24735-z 34362892 PMC8346516

[12] Little EC Camp ER Wang C Watson PM Watson DK Cole DJ. The CaSm (LSm1) Oncogene Promotes Transformation, Chemoresistance and Metastasis of Pancreatic Cancer Cells. Oncogenesis (2016) 5:e182. doi: 10.1038/oncsis.2015.45 26751936 PMC4728675

[13] Watson PM Miller SW Fraig M Cole DJ Watson DK Boylan AM. CaSm (LSm-1) Overexpression in Lung Cancer and Mesothelioma is Required for Transformed Phenotypes. Am J Respir Cell Mol Biol (2008) 38:671–8. doi: 10.1165/rcmb.2007-0205OC PMC2396246 18218995

[14] Hou W Zhang Y. Circ_0025033 Promotes the Progression of Ovarian Cancer by Activating the Expression of LSM4 *via* Targeting miR-184. Pathol Res Pract (2021) 217:153275. doi: 10.1016/j.prp.2020.153275 33285422

[15] Camp RL Dolled-Filhart M Rimm DL. X-Tile: A New Bio-Informatics Tool for Biomarker Assessment and Outcome-Based Cut-Point Optimization. Clin Cancer Res (2004) 10:7252–9. doi: 10.1158/1078-0432.CCR-04-0713 15534099

[16] Hanzelmann S Castelo R Guinney J. GSVA: Gene Set Variation Analysis for Microarray and RNA-Seq Data. BMC Bioinf (2013) 14:7. doi: 10.1186/1471-2105-14-7 PMC3618321 23323831

[17] Bindea G Mlecnik B Tosolini M Kirilovsky A Waldner M Obenauf AC. Spatiotemporal Dynamics of Intratumoral Immune Cells Reveal the Immune Landscape in Human Cancer. Immunity (2013) 39:782–95. doi: 10.1016/j.immuni.2013.10.003 24138885

[18] Rooney MS Shukla SA Wu CJ Getz G Hacohen N. Molecular and Genetic Properties of Tumors Associated With Local Immune Cytolytic Activity. Cell (2015) 160:48–61. doi: 10.1016/j.cell.2014.12.033 25594174 PMC4856474

[19] Yoshihara K Shahmoradgoli M Martinez E Vegesna R Kim H Torres-Garcia W. Inferring Tumour Purity and Stromal and Immune Cell Admixture From Expression Data. Nat Commun (2013) 4:2612. doi: 10.1038/ncomms3612 24113773 PMC3826632

[20] Fang H Guo Z Chen J Lin L Hu Y Li Y. Combination of Epigenetic Regulation With Gene Therapy-Mediated Immune Checkpoint Blockade Induces Anti-Tumour Effects and Immune Response *In Vivo* . Nat Commun (2021) 12:6742. doi: 10.1038/s41467-021-27078-x 34795289 PMC8602287

[21] Kontos F Michelakos T Kurokawa T Sadagopan A Schwab JH Ferrone CR. B7-H3: An Attractive Target for Antibody-Based Immunotherapy. Clin Cancer Res (2021) 27:1227–35. doi: 10.1158/1078-0432.CCR-20-2584 PMC7925343 33051306

[22] Marin-Acevedo JA Dholaria B Soyano AE Knutson KL Chumsri S Lou Y. Next Generation of Immune Checkpoint Therapy in Cancer: New Developments and Challenges. J Hematol Oncol (2018) 11:39. doi: 10.1186/s13045-018-0582-8 29544515 PMC5856308

[23] Qin S Xu L Yi M Yu S Wu K Luo S. Novel Immune Checkpoint Targets: Moving Beyond PD-1 and CTLA-4. Mol Cancer (2019) 18:155. doi: 10.1186/s12943-019-1091-2 31690319 PMC6833286

[24] Peng H Fu YX. The Inhibitory PVRL1/PVR/TIGIT Axis in Immune Therapy for Hepatocellular Carcinoma. Gastroenterology (2020) 159:434–6. doi: 10.1053/j.gastro.2020.06.024 32574623

[25] Charoentong P Finotello F Angelova M Mayer C Efremova M Rieder D. Pan-Cancer Immunogenomic Analyses Reveal Genotype-Immunophenotype Relationships and Predictors of Response to Checkpoint Blockade. Cell Rep (2017) 18:248–62. doi: 10.1016/j.celrep.2016.12.019 28052254

[26] Kelley JR Fraser MM Hubbard JM Watson DK Cole DJ. CaSm Antisense Gene Therapy: A Novel Approach for the Treatment of Pancreatic Cancer. Anticancer Res (2003) 23:2007–13. 12894573

[27] Fraser MM Watson PM Fraig MM Kelley JR Nelson PS Boylan AM. CaSm-Mediated Cellular Transformation is Associated With Altered Gene Expression and Messenger RNA Stability. Cancer Res (2005) 65:6228–36. doi: 10.1158/0008-5472.CAN-05-0650 16024624

[28] Mili D Georgesse D Kenani A. Localization and Role of RAP55/LSM14 in HeLa Cells: A New Finding on the Mitotic Spindle Assembly. Acta Biochim Pol (2015) 62:613–9. doi: 10.18388/abp.2015_1107 26339800

[29] Lee HL Jang JW Lee SW Yoo SH Kwon JH Nam SW. Inflammatory Cytokines and Change of Th1/Th2 Balance as Prognostic Indicators for Hepatocellular Carcinoma in Patients Treated With Transarterial Chemoembolization. Sci Rep (2019) 9:3260. doi: 10.1038/s41598-019-40078-8 30824840 PMC6397294

[30] Kogame M Nagai H Shinohara M Igarashi Y Sumino Y Ishii K. Th2 Dominance Might Induce Carcinogenesis in Patients With HCV-Related Liver Cirrhosis. Anticancer Res (2016) 36:4529–36. doi: 10.21873/anticanres.11000 27630292

[31] Hattori E Okumoto K Adachi T Takeda T Ito J Sugahara K. Possible Contribution of Circulating Interleukin-10 (IL-10) to Anti-Tumor Immunity and Prognosis in Patients With Unresectable Hepatocellular Carcinoma. Hepatol Res (2003) 27:309–14. doi: 10.1016/j.hepres.2003.07.002 14662119

[32] Mao JX Guo WY Guo M Liu C Teng F Ding GS. Acute Rejection After Liver Transplantation Is Less Common, But Predicts Better Prognosis in HBV-Related Hepatocellular Carcinoma Patients. Hepatol Int (2020) 14:347–61. doi: 10.1007/s12072-020-10022-4 32140981

[33] DeNardo DG Coussens LM. Inflammation and Breast Cancer. Balancing Immune Response: Crosstalk Between Adaptive and Innate Immune Cells During Breast Cancer Progression. Breast Cancer Res (2007) 9:212. doi: 10.1186/bcr1746 17705880 PMC2206719

[34] Zhuang H Zhou Z Zhang Z Chen X Ma Z Huang S. B3GNT3 Overexpression Promotes Tumor Progression and Inhibits Infiltration of CD8(+) T Cells in Pancreatic Cancer. Aging (Albany NY) (2020) 13:2310–29. doi: 10.18632/aging.202255 PMC7880340 33316775

[35] Kobayashi Y Lim SO Yamaguchi H. Oncogenic Signaling Pathways Associated With Immune Evasion and Resistance to Immune Checkpoint Inhibitors in Cancer. Semin Cancer Biol (2020) 65:51–64. doi: 10.1016/j.semcancer.2019.11.011 31874279

[36] Zhu S Ma AH Zhu Z Adib E Rao T Li N. Synergistic Antitumor Activity of Pan-PI3K Inhibition and Immune Checkpoint Blockade in Bladder Cancer. J Immunother Cancer (2021) 9:e002917. doi: 10.1136/jitc-2021-002917 34725212 PMC8562536

[37] Liu X Yin M Dong J Mao G Min W Kuang Z. Tubeimoside-1 Induces TFEB-Dependent Lysosomal Degradation of PD-L1 and Promotes Antitumor Immunity by Targeting mTOR. Acta Pharm Sin B (2021) 11:3134–49. doi: 10.1016/j.apsb.2021.03.039 PMC8551420 34745852

